# Tyrosine phosphorylation of Grb14 by Tie2

**DOI:** 10.1186/1478-811X-8-30

**Published:** 2010-10-25

**Authors:** Celina Sturk, Daniel J Dumont

**Affiliations:** 1 Molecular and Cellular Biology Research, Sunnybrook Research Institute, Sunnybrook Health Sciences Centre, Toronto, ON, Canada; 2 Department of Medical Biophysics, University of Toronto, ON, Canada

## Abstract

**Background:**

Growth factor receptor bound (Grb) proteins 7, 10 and 14 are a family of structurally related multi-domain adaptor proteins involved in a variety of biological processes. Grb7, 10 and 14 are known to become serine and/or threonine phosphorylated in response to growth factor (GF) stimulation. Grb7 and 10 have also been shown to become tyrosine phosphorylated under certain conditions. Under experimental conditions Grb7 is tyrosine phosphorylated by the Tie2/Tie-2/Tek angiogenic receptor tyrosine kinase (RTK). Furthermore, Grb14 has also been shown to interact with Tie2, however tyrosine phosphorylation of this Grb family member has yet to be reported.

**Results:**

Here we report for the first time tyrosine phosphorylation of Grb14. This phosphorylation requires a kinase competent Tie2 as well as intact tyrosines 1100 and 1106 (Y1100 and Y1106) on the receptor. Furthermore, a complete SH2 domain on Grb14 is required for Grb14 tyrosine phosphorylation by Tie2. Grb14 was also able to become tyrosine phosphorylated in primary endothelial cells when treated with a soluble and potent variant of the Tie2 ligand, cartilage oligomeric matrix protein (COMP) Ang1.

**Conclusion:**

Our results show that Grb14, like its family members Grb7 and Grb10, is able to be tyrosine phosphorylated. Furthermore, our data indicate a role for Grb14 in endothelial signaling downstream of the Tie2 receptor.

## Background

Signal transduction pathways encompass a series of highly coordinated events involving numerous proteins, varying in both structure and function. Adaptor proteins play a pivotal role in such molecular networks by allowing formation of protein complexes via interactions involving their non-catalytic binding domains. The Growth factor Receptor Bound (Grb) adaptor proteins are a group of structurally similar proteins beginning to emerge as key players in a number of cellular functions including cell metabolism, cell survival and cell migration.

The Grb family is made up of three members, Grb7,10 and 14. All family members harbor an amino-terminal proline rich region (PRR), a putative Ras associating (RA) domain, a pleckstrin homology (PH) domain and a carboxyl-terminal SH2 domain. Furthermore, unique to this family of proteins is a novel interaction region, the BPS (for Between PH and SH2) domain. To date the BPS domain has been shown to play a role in certain Grb/receptor interactions including those involving the activated Insulin receptor (IR) and Insulin-like Growth Factor Receptor [1].

While all three family members were originally cloned in screens using the EGFR as bait, as with most other SH2 containing proteins it was quickly discovered that this family of adaptors binds a number of other receptor and non-receptor proteins. Included in these is the angiogenic Tie2/Tie-2/Tek receptor tyrosine kinase (RTK). Tie2 is a RTK found primarily on the surface of endothelial cells. Mouse molecular models have shown an essential role for this receptor in aspects of angiogenesis. Tie2 deficient mice present with a plethora of vascular abnormalities including a lack of proper sprouting and remodeling of the primitive vessel network [2,3]. In the adult, Tie2 has been implicated in both normal and pathological states including wound healing, follicular development, diabetic retinopathy and tumorigenesis.

Tie2 belongs to the Tie family of proteins. While the only other family member, Tie/Tie-1, remains an orphan receptor, a family of ligands known as the angiopoietins (Angs) have been shown to play a role in Tie2 biology. Currently, Ang1 remains the best characterized of the angiopoietins and is believed to be the main activating ligand for Tie2. It has been shown to activate the Tie2 receptor and contribute to such functions as endothelial cell survival and migration [4].

Jones et al. initially demonstrated Grb7 and Grb14 interactions with Tie2 in a yeast two-hybrid screen using cDNAs derived from embryonic mouse heart and lung tissue [5]. Overexpression studies further demonstrated that the Grb7/Tie2 interaction was mediated by a multidocking site, tyrosine 1100, on Tie2. Furthermore, Grb7 becomes tyrosine phosphorylated in the presence of a kinase competent Tie2 receptor.

The Grb family of proteins are known to be phosphorylated on serine, threonine and tyrosine residues, although the functional significance of these phosphorylations are still unclear in most cases. Grb7 is phopshorylated on serine and threonine residues in both quiescent and growth factor (GF) stimulated cells, although GF stimulation does not appear to alter Grb7 phosphorylation state [6,7]. Grb7 has also been shown to be tyrosine phosphorylated by a number of different kinases, including FAK at sites of focal adhesion and,in the presence of certain GFs such as EGF, and ephrinB1 and in the presence of receptors such as Tie2, Ret and HER2/erbB-2 [5,6].

In contrast to Grb7, both Grb10 and Grb14 possess basal serine phosphorylation which can be further induced by GF stimulation [8-11]. Grb10 has also been shown to be tyrosine phosphorylated in insulin signaling and in the presence of active Tec [12-14]. To date, there are no previous reports of Grb14 tyrosine phosphorylation, although it has been predicted to occur under certain conditions given its interaction with numerous RTKs and its similar structure to Grb7 and 10.

Here we report for the first time Grb14 tyrosine phosphorylation. This phosphorylation appears to be dependent on the presence of Tie2 kinase activity and appears to involve tyrosine residues 1100 and 1106 of the Tie2 RTK as well as an intact SH2 domain on Grb14.

## Results

### Grb14 becomes tyrosine phosphorylated in the presence of Tie2

**Figure 1:**
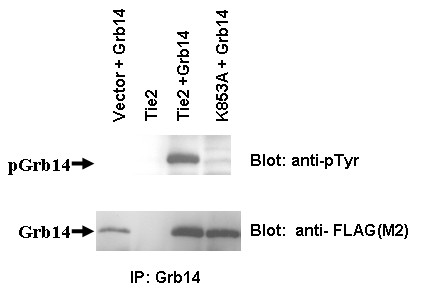
**Grb14 is tyrosine phosphorylated in the presence of Tie2**. 293T kidney epithelial cells were transfected with Grb14 either alone or with the wild type receptor (Tie2) or a kinase inactive mutant of Tie2 (K853A). Cell lysates from these transfections were then subjected to immunoprecipitation using an anti-Grb14 antibody and blotted for phospho-tyrosine (4G10) or anti-FLAG to check for Grb14 amount precipitated.

While both Grb7 and Grb10 have been reported to be tyrosine phosphorylated [5,6,12-14], Grb14 has only been found to become phosphorylated on serine and threonine residues [9,10]. Because Tie2 is a known tyrosine kinase, we set out to determine whether or not Grb14 could become tyrosine phosphorylated in the presence of this receptor. 293T kidney epithelial cells were transfected with Grb14 either alone or with the wild type receptor (WT) or a kinase inactive mutant of Tie2, K853A. Cell lysates from these transfections were then subjected to immunoprecipitation using an anti-Grb14 antibody and blotted for phospho-tyrosine (Figure 1). Grb14 becomes tyrosine phosphorylated in the presence of the wild type receptor (WT) but not vector or K853A. This shows for the first time that Grb14 can be tyrosine phosphorylated and in this case, phosphorylation is dependent on the presence of the Tie2 kinase.

### Y1100 and Y1106 on Tie2 are important for Grb14 tyrosine phosphorylation

**Figure 2:**
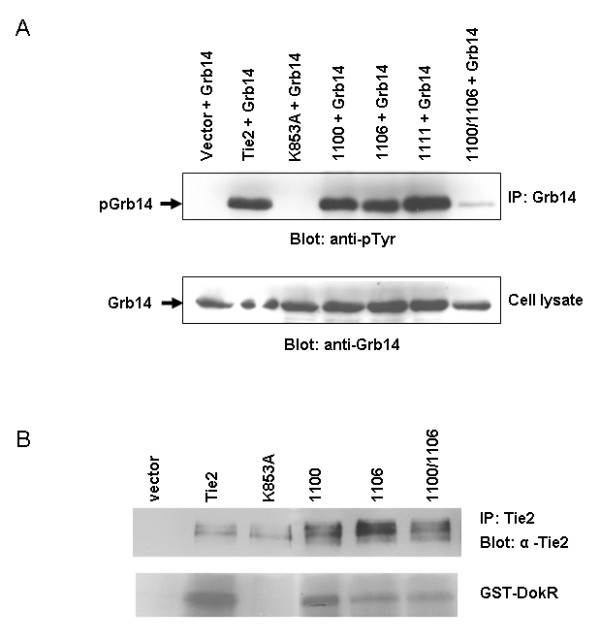
**Y1100 and Y1106 on Tie2 are important for Grb14 tyrosine phosphorylation**. HEK293T cells were transfected with Grb14 alone or in combination with wild type Tie2, the kinase deficient Tie2 (K853A) or one of the Tie2 tyrosine mutants (Y1100F, Y1106F, Y1111F, Y1100/1106F). A) Lysates (100 μg) from these cells were immunoprecipitated using an antibody specific for Grb14, run out on an SDS PAGE gel and blotted for phosphotyrosine (top panel). Cell lysates (20 μg) were also run out on an SDS PAGE gel and subjected to Western blotting using an anti-Grb14 antibody as a control (bottom panel). B) Lysates were immunoprecipitated with an anti-Tie2 antibody and then either subjected to Western Blot using anti-Tie2 antibodies (top panel) or incubated in a kinase reaction with set amounts of glutathione-Sepharose purified GST-Dok-P^ΔPH ^and ^32^P-labeled ATP (bottom panel).

RTKs function by autophosphorylating on tyrosine residues in their intracellular domain upon activation, often providing binding sites to various proteins containing phospho-tyrosine binding domains. Grb14 contains a C-terminal SH2 domain and is believed to bind to Tie2 via one of the receptor's phospho-tyrosine residues. We used a number of Tie2 tyrosine to phenylalanine point mutants in order to determine whether or not mutation of any of these tyrosine residues on Tie2 altered Grb14 phosphorylation. 293T cells were transfected with Grb14 alone or in combination with wild type Tie2, the kinase deficient Tie2 (K853A) or one of the Tie2 tyrosine mutants (Y1100F, Y1106F, Y1111F, Y1100/1106F). While mutation of any one of the Tie2 tyrosine residues did not appear to alter Grb14 phosphorylation significantly, when both Y1100 and Y1106 were mutated on the same receptor (Y1100/1106) there was a marked decrease in Grb14 tyrosine phosphorylation (Figure 2A). This suggests that Y1100 and Y1106 on Tie2 play a role in Grb14 mediated signal transduction downstream of this receptor.

Previous studies have shown that mutation of receptor tyrosine residues to phenylalanine (such as in our Tie2 mutants) can affect kinase activity of the receptor. In order to determine if the substitution of Y1100 or 1106 results in a decreased in kinase activity, we carried out a kinase assay to compare the kinase activity of the Y1100F, Y1106F and Y1100/1106F mutants to that of the wild type receptor using a GST Dok-R fusion protein as substrate. Dok-R is a docking protein that becomes tyrosine phosphorylated downstream of Tie2 *in vivo *making it a biologically relevant choice for a substrate. The Dok-R construct used herein lacks the insoluble PH domain to facilitate purification and will be subsequently referred to as C (described previously in [15]. An equal amount of receptor was immunoprecipitated from 293T cells expressing Tie2WT, K853A or one of the Tie2 mutants (Y1100F, Y1106F or Y1100/1106F) and incubated in a kinase reaction with set amounts of glutothione-Sepharose purified GST-Dok-P^ΔPH ^and ^32^P labeled ATP. Figure 2B is a representative blot of two separate experiments demonstrating ability of Y1100/1106F to phosphorylate GST-Dok-P^ΔPH^. These data show that while there appears to be a slight decrease in Y1100/1106F kinase activity when compared to Tie2WT, Y1100/1106F activity is comparable to that of the Y1106F mutant. Recall from Figure 2A that Grb14 was phosphorylated in the presence of Y1106F mutant to similar levels as when in the presence of the wild type receptor indicating that the level of kinase activity of the Y1106 mutant is adequate for phosphorylation of Grb14. By inference, therefore, we believe it is unlikely that the decrease in kinase activity seen here for Y1100/1106F is the cause of the dramatic decrease in Grb14 tyrosine phosphorylation observed in our overexpression studies.

### Grb14 SH2 domain is important for Grb14 tyrosine phosphorylation

**Figure 3:**
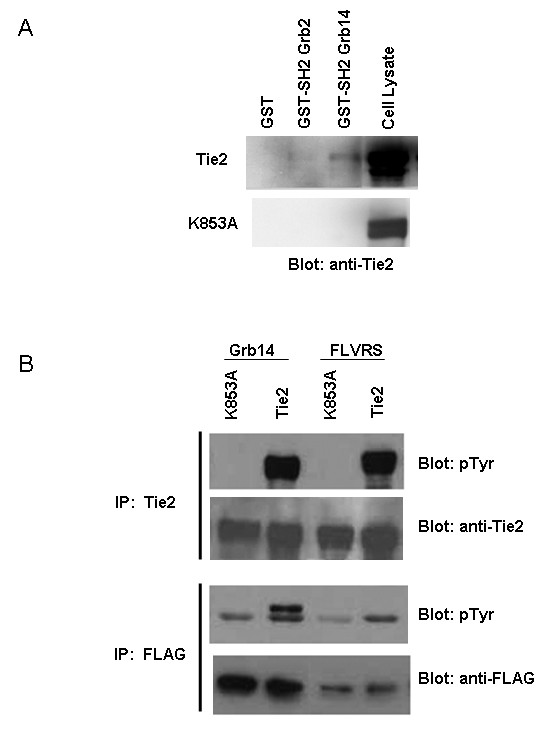
**The SH2 domain is important for Grb14 tyrosine phosphorylation**. A) HEK 293T cells were transfected with Tie2, either WT or the kinase inactive K853A mutant. Lysates were incubated with immobilized GST alone, GST-SH2 domains from Grb2 (control) or Grb14 (as indicated). Resulting complexes were resolved by SDS-PAGE and immunoblotted with anti-Tie2 antibodies. B) HEK 293T cells were co-transfected with Tie2 (WT or kinase inactive) and FLAG tagged Grb14 (WT or the FLVRS mutant). Lysates subjected to immunoprecipitation by either Tie2 or FLAG antibodies and resolved by SDS-PAGE. Western blotting with indicated antibodies was then carried out.

Both SH2 and BPS domains of the Grb family members have been shown to mediate interactions with various receptor and non-receptor proteins. In order to investigate whether or not the SH2 domain of Grb14 is able to bind Tie2 we performed an *in vitro *pull down assay using the full length Tie2 receptor and the SH2 domain of Grb14. Grb14 and Grb2 (as a positive control) SH2 domains were purified from *E. coli *and immobilized on glutathione-Sepharose beads. GST-SH2 domains were then incubated with lysates from HEK293T cells transiently expressing either WT Tie2 or the kinase inactive K853A mutant. The SH2 domains of both Grb2 and Grb14 were both able to pull down WT Tie2, but not K853A (Figure 3A) indicating the ability of the Grb14 SH2 domain to interact with Tie2. The increased ability of Grb14 to pull-down Tie2 as compared to Grb2 may reflect a greater interaction with Tie2. Cell lysate from transfected cells was analyzed to confirm transfection and protein expression in 293T cells.

Mutation of the SH2 domain FLVRS motif has been shown to disrupt SH2 domain function. We generated an Arg to Lys (R646K) mutation in the Grb14 SH2 domain within this conserved FLVRS motif in order to determine the effect of disrupting Grb14 SH2 function on Grb14 tyrosine phosphorylation. HEK293T cells were transfected with WT Tie2 or the K853A mutant along with either WT Grb14 or our Grb14FLVRS mutant. Lysates from these cells were immunoprecipitated with anti-FLAG antibody and subjected to Western blot using a phospho-tyrosine specific antibody (Figure 3B). Figure 3B shows that mutation of the Grb14 FLVRS motif abolishes tyrosine phosphorylation of Grb14 suggesting the functionality of this domain must be intact for Tie2 mediated Grb14 tyrosine phosphorylation. Blots were stripped and reprobed using anti-Tie2 and anti-FLAG antibodies as controls. Together these results suggest a role for the Grb14 SH2 domain in Tie2 mediated Grb14 signaling.

### Grb14 phosphorylation in endothelial cells

**Figure 4:**
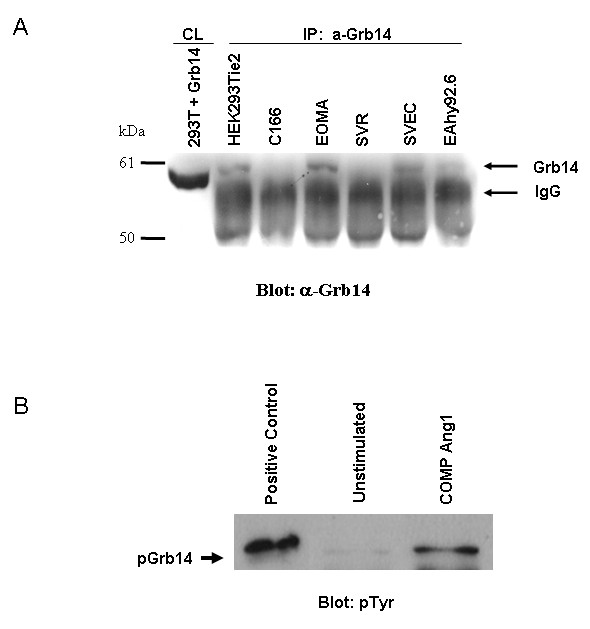
**Tyrosine phosphorylation of Grb14 in endothelial cells**. A) Lysates from C166, EOMA, SVR, SVEC and EAhy92.6 endothelial cell lines as well as an HEK293 cell line that stably expresses the full-length Tie2 receptor (HEK293Tie2) were immunoprecipitated and then subjected to Western Blotting using an antibody specific for Grb14. Cell lysate from 293T cells overexpressing Grb14 was also run as a positive control. B) Human umbilical endothelial cells (HUVEC) were either left untreated or stimulated with the soluble and potent variant of the Tie2 ligand, cartilage oligomeric matrix protein (COMP) Ang1. Cell lysates were immunoprecipitated with anti-Grb14 and subjected to SDS PAGE and Western blotted for phosphotyrosine with 4G10 antibody. The positive control lane was protein sample derived from Grb14/Tie2Y1111 transfected 293T cells

A yeast-two hybrid screen using cDNA derived from murine embryonic heart and lung tissue and the intracellular portion of the Tie2 receptor initially identified Grb14 as a binding partner of Tie2 [5]. To date, Tie2 has been shown to be found predominantly in endothelial cells. In order to verify that Grb14 is a potentially biologically relevant player in the Tie2 signaling pathway, we examined for the presence of Grb14 in a number of endothelial cell lines. Four murine (C166, EOMA, SVR and SVEC) and one human (EAhy92.6) endothelial cell lines as well as an HEK293 cell line that stably expresses the full-length Tie2 receptor (HEK293Tie2) were immunoprecipitated and then subjected to Western Blotting using an antibody specific for Grb14 (Figure 4A). Cell lysate from 293T cells overexpressing Grb14 was also run as a positive control for Grb14. The results show that Grb14 is present in three of the five endothelial cell lines examined (EOMA, SVEC and EAhy92.6). These results were confirmed by RT-PCR (data not shown). This data demonstrates that Grb14 is in fact present in endothelial cells and therefore is a reasonable candidate for a player in the Tie2 signaling pathway.

Angiopoietin-1 (Ang1) is a known ligand of Tie2 in endothelial cells inducing the Tie2 signalling pathway. In order to verify whether or not endogenous Grb14 is able to be tyrosine phosphorylated in endothelial cells, we stimulated HUVEC cells with a soluble and potent variant of the Tie2 ligand, cartilage oligomeric matrix protein (COMP)-Ang1 [16]. Cell lysate from either unstimulated cells or cells treated with COMP-Ang1 was run on a gel and probed for phosphotyrosine. Lysate from 293T cells transfected with Y1111F and Grb14 was run as a positive control for Grb14 phosphorylation. Figure 4B shows a tyrosine phosphorylated protein running at the same size as our positive control in the COMP-Ang1 sitmulated lane, but to a much lesser extent in the unstimulated sample. This data suggests that Grb14 is phosphorylated in endothelial cells upon stimulation with the Ang1 variant, COMP-Ang1.

## Discussion

Although the Grb family of proteins was described for the first time over a decade ago, still much has to be learned about the role of these adaptors in various signaling pathways. Most of what we know about these proteins has come from binding studies and elucidation of the various domains involved in these interactions. Grb7, 10 and 14 have been shown to bind a number of receptors and intracellular proteins primarily via SH2 mediated interactions, although the importance of the BPS domain is also becoming apparent.

Grb14 was previously identified in a yeast two-hybrid screen as a binding partner for the endothelial receptor Tie2 [5]. Tie2 is an angiogenic RTK involved in numerous aspects of endothelial biology such as cell migration, cell survival and tubule formation. We have now been able to show that Grb14 is endogenous to endothelial cells further supporting a role for Grb14 in endothelial cell signaling. Furthermore, the results reported herein are the first to describe tyrosine phosphorylation of Grb14. This phosphorylation appears to require a kinase competent Tie2 and tyrosines 1100 and 1106 on the receptor.

Phosphorylation is the most widespread post-translational protein modification in cell signaling [17]. Phosphorylation of a protein can modulate its behavior in a multitude of ways including its function, localization, half life and binding to other molecules [18]. In eukaryotes, phosphorylation is typically 'carried out' on serine, threonine or tyrosine residues.

Since there discovery, Grb family members have been shown become phosphorylated under a number of various conditions. The role of this phosphorylation, however, remains somewhat enigmatic. All family memebers (Grb7, 10 and 14) have been shown to possess both basal and growth factor induced serine phosphorylation (reviewed in Holt, 2005 [19]). Grb10 and Grb7 have also been shown to be tyrosine phosphorylated. More specifically, Grb10 and Grb7 tyrosine phosphorylation has been previously implicated in signaling downstream of endothelial receptors. Grb10 has been shown to be tyrosine phosphorylated in endothelial cells by VEGF [20]. Our lab has also previously shown that Grb7 becomes tyrosine phosphorylated in the presence of the endothelial receptor Tie2 [5]. Taken together, these data supports a role for Grb proteins in endothelial biology.

The current study suggests that Grb14 tyrosine phosphorylation requires a kinase competent Tie2 receptor. In a separate experiment, Grb14 was not tyrosine phosphorylated in the presence of EGFR suggesting some specificity for the Tie2 kinase (data not shown). Whether or not Grb14 is a direct substrate of the Tie2 kinase activity, or rather is phosphorylated by an alternate kinase recruited to this receptor, remains to be seen.

Grb proteins do not possess intrinsic kinase activity, but have been shown to bind a number of receptor and non-receptor kinases (reviewed in [7,19,21]. In insulin signaling, Grb10 serine phosphorylation appears to involve the Pi3K and MAPK signaling pathways, while PKCζ seems to play a role in Grb14 phosphorylation [11,22]. Grb proteins have been found to bind a number of tyrosine kinases (reviewed in Holt and Siddle, 2005 [19]). Interestingly, in the case of insulin signaling, although Grb10 and Grb14 bind the IR, they do not appear to be direct substrates for the IR tyrosine kinase activity [13,23]. Instead, at least with respect to Grb10, Src/Fyn kinases were suggested to be responsible. In general, how and why Grb proteins are phosphorylated remains to be determined in most cases.

In our studies, Grb14 tyrosine phosphorylation was abolished upon mutation of Y1100 and Y1106 to phenylalanine in the receptor double mutant Tie21100/1106, suggesting these two residues may play a role in Grb14 tyrosine phosphorylation downstream of Tie2. This should perhaps not be surprising given that there is emerging evidence that the Grb family members may bind their target proteins via two separate regions, the SH2 and BPS domains (Reviewed in Holt and Siddle, 2005 [19]).

This is particularly evident in the case of insulin signaling where both SH2 and BPS domains of Grb 7, 10 and 14 have been shown to bind the IR [19]. Specifically, structural studies have shown that the Grb14 SH2 and BPS domains bind phosphorylated tyrosine residues within the IR activation loop [24]. Whether tyrosines 1100 and 1106 bind Grb14 directly or play a more indirect role in Grb14 tyrosine phosphorylation, however, remains to be determined.

It is interesting to note that in comparison with the other Grb family members, Grb14 has been shown to interact with a relatively small number of receptors. In accordance with this observation, we have found *in vivo *binding studies of Grb14 and Tie2 have been particularly challenging (data not shown). This may be explained, at least in part, by structural studies which have shown that Grb14 may have more difficulty binding to phosphotyrosine containing ligands due to the presence of a non-glycyl residue at the end of the BC loop and the lack of a P+3 binding pocket in the SH2 domain [25].

Further analysis of Grb14 tyrosine phosphorylation will no doubt provide information that may help elucidate the role of this protein in Tie2 signaling. Mapping the tyrosines on Grb14 which become tyrosine phosphorylated may give us a clue as to what other proteins bind and are involved in Grb14 signaling. Tyr67 on Grb10 was identified as major site of phosphorylation in response to insulin signaling. However, this site is not conserved in Grb7 and 14. Interestingly, mutation of this site increased affinity of Grb10 for IR. This raises the possibility that tyrosine phosphorylation may be involved in terminating Grb signaling at the receptor level. Alternatively, it may suggest that Grb tyrosine phosphorylation recruits this adaptor for involvement in non-receptor mediated signaling pathways. Further understanding of these sorts of post-translational modifications seen in the Grb family will no doubt shed considerable insight into their biological role.

## Conclusions

Grb14 becomes tyrosine phosphorylated upon Tie2 activation and this tyrosine phosphorylation is dependent upon tyrosine residues 1100 and 1106 of Tie2.

## Methods

### Expression vectors

The cDNAs representing wild type (WT), kinase-inactive (K853A) and Y1111F Tie2/Tie-2 in pcDNA3.1 have been described previously [5]. The cDNA encoding Grb14 was a kind gift from Roger Daly. The cDN encoding the full length Grb14 [10] was used as a template to generate an arginine-to-lysine point mutation with the QuickChange site-directed mutagenesis kit (Stratagene). Mutation was confirmed by sequencing. GST fusion proteins were described previously [5].

### Production of GST fusion proteins

GST fusion proteins were prepared from *Escherichia coli *using standard procedures. Recombinant proteins were immobilized on glutathione-Sepharose beads (Amersham) at 4°C for 30 minutes and analyzed by SDS-polyacrylamide gel electrophoresis followed by Coomassie Blue staining. Bovine serum albumin standards were used as a comparison to estimate protein concentration.

### Cell Culture and Antibodies

Human umbilical vein endothelail (HUVEC) cells C166, SVR, SVEC and HEK293T and HEK293Tie2 (a gift of Fu-Kuen Lin, Amgen) cells were grown on 10-cm plates in Dulbecco's modified Eagle's medium (DMEM) (Sigma) supplemented with 10% FBS, 1% penicillin, 1% streptomycin, and 200mM L-glutamine. EA.hy926 cells were further supplemented with hypoxanthine, aminopterin and thymidine (Sigma) and HEK293Tie2 cells were further supplemented with 250 mg/ml G418 (Life Technologies, Inc.), and 100 nM methotrexate (Sigma). Antibodies used were as follows: polyclonal anti-Tie2 C-20 (Santa Cruz), monoclonal anti-phosphotyrosine 4G10 (Upstate Biotechnology Inc.), monoclonal anti-Tie2 (Pharmingen), monoclonal anti-Flag M2 (Sigma), polyclonal phospho-Tie2 (992) (Cell Signaling Technology). GST mixing experiments, coimmunoprecipitation experiments, and Western blotting procedures have been previously described [5,15]. COMP-Ang1 was a kind gift of GY Koh, Korea.

### Transfection Procedures

HEK293T cells were cultured to ~75% confluency in 10-cm cell culture plates and transfected with 2 μg DNA using the lipofectamine reagent (Invitrogen Life Technologies) and according to manufactures protocol. Cells were harvested after 48 h.

### In Vitro Kinase Assay

Kinase assays were performed as follows: Cell lysates were immunoprecipitated with anti-Tie2 antibodies for 2 h at 4°C. Immunoprecipitates were washed 3x in PLC lysis buffer (without sodium fluoride or sodium pyrophosphate) [15] and 2x in kinase buffer (2 mM MnCl_2 _+ 50 mM HEPES pH 7.5 + 10 mM MgCl_2 _+ 0.2 mM dithiothreitol) and then incubated with 4 μg of either GST or GST-Dok-P^ΔPH ^as substrate including 40 μCi of [g-^32^P]ATP (Amersham Biosciences), 20 μM ATP (Amersham Biosciences) for 30 min at 30°C [26]. Kinase reactions were stopped by the addition of 2x SDS-containing sample buffer and boiled for 10 min. Immunoprecipitates were electrophoresed and half the gel was used to resolve Tie2/Tie-2 expression by Western analysis using anti-Tie-2 antibodies while the other half was dried and exposed to phosphorimager analysis and quantification (ImageQuant). The representative values of Tie2 kinase activity were reflected as the value of GST-Dok-P^ΔPH ^phosphorylation over the amount of immunoprecipitated Tie2 in each of the samples. All experiments were performed twice or more times with similar results.

## Competing interests

The authors declare that they have no competing interests.

## Authors' contributions

CS performed all the experiments and wrote the manuscript. DD revised the manuscript and provided final approval for publication. All authors have read and approved the final manuscript.
